# Ribonucleoprotein Complexes That Control Circadian Clocks

**DOI:** 10.3390/ijms14059018

**Published:** 2013-04-25

**Authors:** Dongni Wang, Xiaodi Liang, Xianyun Chen, Jinhu Guo

**Affiliations:** Key Laboratory of Gene Engineering of the Ministry of Education, State Key Laboratory of Biocontrol, School of Life Sciences, Sun Yat-sen University, Guangzhou 510275, China; E-Mails: wdn001@sina.cn (D.W.); sodylong@sina.com (X.L.); chenxianyun3000@sina.com (X.C.)

**Keywords:** ribonucleoprotein, circadian clock, transcription, translation, post-transcription

## Abstract

Circadian clocks are internal molecular time-keeping mechanisms that enable organisms to adjust their physiology and behavior to the daily surroundings. Misalignment of circadian clocks leads to both physiological and health impairment. Post-transcriptional regulation and translational regulation of circadian clocks have been extensively investigated. In addition, accumulating evidence has shed new light on the involvement of ribonucleoprotein complexes (RNPs) in the post-transcriptional regulation of circadian clocks. Numerous RNA-binding proteins (RBPs) and RNPs have been implicated in the post-transcriptional modification of circadian clock proteins in different model organisms. Herein, we summarize the advances in the current knowledge on the role of RNP complexes in circadian clock regulation.

## 1. Introduction

Due to the daily self-rotation of the Earth, environmental factors (e.g., light, temperature, moisture, *etc.*) display cyclic changes with a period of approximately 24 h. The cycling feature of these factors has fundamental influences on organism health and survival. Most organisms, from single-celled to complex organisms, have evolved internal circadian clock systems that operate in concert physiologically and behaviorally, which enable these organisms to adapt to the environment [1]. A number of representative species across different kingdoms are employed as models in circadian clock studies, including cyanobacteria (*Synechococcus elongatus*); filamentous fungi (*Neurospora crassa*); unicellular algae (*Gonyaulax polyedra*); coral (*Acropora digitifera*); insects, such as fruit flies (*Drosophila melanogaster*), honeybees (*Apis mellifera*) and monarch butterflies (*Danaus plexippus*); plants (*Arabidopsis thaliana*); mollusks (*Bulla gouldiana*); fish; birds; rodents and humans [2–8].

**Figure 1:**
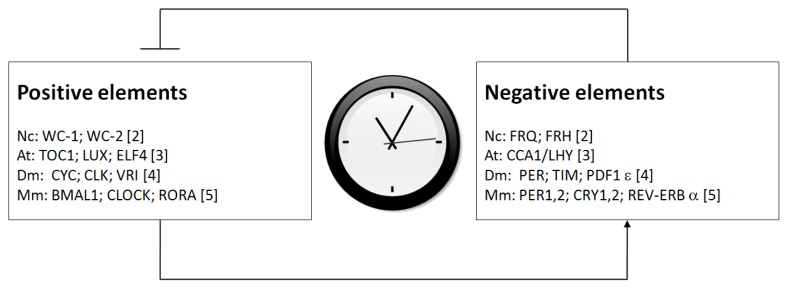
The components of circadian clock oscillators. Circadian clock oscillators are composed of positive elements and circadian clock proteins. The positive elements can bind to the promoter regions of the clock genes and mediate the transcription. Circadian clock proteins feed back to repress the functions of positive elements. The positive and negative elements together constitute negative feedback loops at transcription-translational levels. The species names are abbreviated as Nc: *Neurospora crassa*; Mm: mammals; Dm: *Drosophila melanogaster*; At: *Arabidopsis thaliana*. The circadian input and output are not shown.

At the molecular level, eukaryotic circadian clocks consist of positive and negative components which comprise a set of interplaying clock proteins that act in a highly conserved mechanism. The positive elements are transcription factors that activate transcription of the clock genes. In turn, the clock genes encode negative elements that repress the function of the positive elements. After proteolytic degradation of the clock proteins, the positive elements bind to the promoters of clock genes and re-activate their transcription. Therefore, positive and negative elements constitute negative feedback loops to constitute the circadian oscillators to generate molecular and cellular rhythms, at the transcriptional and translational levels (Figure 1). For instance, in *Neurospora*, the positive elements are WHITE COLLAR 1 (WC-1) and WC-2, and the negative element is FREQUENCY (FRQ) [2]. In mammals, including humans, the positive elements include BMAL1, CLOCK and RORA, and the negative elements include period proteins (PER1, 2), cryptochrome proteins (CRY1, 2) and REV-ERBα [3]. The circadian clocks operate non-orthologously in a highly conserved manner through transcriptional/translational negative feedback loops, despite phylogenetic differences between circadian clock genes in different species. The positive and negative elements in different organisms are listed in Figure 1. The positive elements also rhythmically drive the expression of clock-controlled genes (*ccg*s) as circadian output, at the transcriptional level.

Post-transcriptional regulation is responsible for the regulation in mRNA levels, protein levels as consequence, and diversity of protein isoforms. In addition to transcriptional and translational regulation, epigenetic, post-transcriptional and post-translational controls are emerging as crucial modulators of circadian clocks [9,10]. Though the core circadian system has concentrated on transcriptional control, it has been apparent that substantial regulation is achieved after transcription. In eukaryotes, approximately 1%–10% genes are subjected to circadian control directly or indirectly [6,11]. Koike *et al*. reported in a genome-wide study that only ~1/5 of the mRNAs that display rhythmicity in abundance are directly driven by transcription, which suggests that post-transcriptional mechanisms including RNA splicing, polyadenylation, or mRNA stability are essential layers for generation of gene expression rhythmicity [12–16]. Ribonucleoprotein (RNP) complexes play important roles in all aspects of mRNA processing, surveillance, metabolism and turnover at the post-transcriptional level [17,18]. Recently, a number of ribonucleoproteins have been implicated in circadian clocks by regulation of transcription, transcript processing, transport, surveillance and turnover of the circadian gene transcripts [19–25]. Herein, we review the recent findings on post-transcriptional regulation of circadian clocks by RNP complexes.

## 2. Regulation of mRNA Splicing

Alternative splicing is a major cause of mRNA variability and protein diversity in eukaryotes. The process of intron excision occurs in a RNP complex known as a spliceosome. The spliceosome is one of the largest complexes in the cell, and it is composed of ~200 snRNPs and five snRNAs [26]. Accumulating data suggest that mRNA splicing plays an important role in controlling circadian clock. However, whether splicing is under the control of a circadian clock is lacking substantial evidence.

In *Drosophila*, modulation of circadian gene expression might contribute to the seasonal adaption of activity patterns. *Drosophila* exhibits increased mid-day activity on cold days and is more active during the cooler nighttime on hot days. The *Drosophila* clock gene *Per* contains intron 8 (dmpi8) in its 3′UTR region, and perturbation of dmpi8 splicing leads to increased accumulation of *per* transcripts, possibly due to an alteration in the stability of the mRNA [27,28]. Splicing of dmpi8 is inhibited upon light exposure or temperature increase. In addition, it is temporally controlled by the circadian clock mutation in no-receptor potential-A (norpA), which encodes phospholipase C (PLC/NORPA), and has been reported to cause the over-accumulation of spliced *per* transcripts and an advanced evening activity profile [27,28].

The circadian clock has a property, which enables to maintain its periodicity in spite of daily changes of environmental temperature, which is defined as temperature compensation [29–32]. In *Neurospora*, the clock genes *wc-2* and *frq* express splice variants; the splicing of *frq* has been studied extensively [29–36]. Mutation of *wc-2* at 3484 nt, which is located in the midst of a lariat consensus sequence, led to decrease in *frq* mRNA and FRQ protein levels in the dark [33]. *frq* can be transcribed into eight splice variants in total, and these transcripts can be classified into two groups according to whether they harbor the 6th intron (I-6) [33,37]. The transcripts with I-6 encode a large-sized FRQ protein isoform (l-FRQ, large FRQ), while the transcripts without I-6 encode a small-sized FRQ isoform (s-FRQ, small FRQ). s-FRQ lacks the *N*-terminal fragment (99aa) due to the use of a different initiation codon than that of l-FRQ [29,30,33]. The expression of l-FRQ and s-FRQ is controlled by temperature. The ratio of s-FRQ/l-FRQ is increased at a low temperature and decreased at a high temperature. The expression of l-FRQ inversely correlates with that of s-FRQ [31–34]. Removal of either s-FRQ or l-FRQ impairs temperature compensation of the circadian clock [30,35], which demonstrates that splicing of *frq* mRNA is critical for the clock temperature compensation in *Neurospora*.

In the *Arabidopsis* circadian clock, the core gene *CCA1* produces a splice variant *CCA1β* that is associated with low temperature responses [38].

A few of splicing factors have been identified to regulate the expression of clock genes and clock phenotypes as a consequence. In *Arabidopsis*, SNW/Ski-interacting protein (SKIP), a splicing factor and component of the spliceosome, is involved in the post-transcriptional regulation of circadian clock genes in *Arabidopsis*. SKIP interacts with the spliceosomal splicing factor Ser/Arg-rich protein 45 to regulate pre-mRNA splicing of clock genes, such as *PSEUDORESPONSE REGULATOR7 (PRR7)* and *PRR9*. Mutation in *SKIP* lengthens the circadian period in a temperature-sensitive manner and affects the circadian clock’s response to light input and the sensitivity of the circadian clock to light resetting. In comparison with the wild type gene, the *skip-1* mutant exhibits a comparable circadian period at 27 °C but a ~3.5 h longer period at 17 °C, which suggests that SKIP is also required for circadian clock temperature compensation [39]. These facts point to a possibility that mRNA splicing might be a common mechanism in controlling the temperature compensation of circadian clocks in different lineages.

*Arabidoposis* SPLICEOSOMAL TIMEKEEPER LOCUS1 (STIPL1), a putative RNA-binding protein, is a homolog of the spliceosomal proteins TFP11 (*Homo sapiens*) and Ntr1p (*Saccharomyces cerevisiae*) [40]. Mutation in STIPL1 results in a long circadian period. STIPL1 regulates the splicing of the mRNAs of clock genes *CCA1*, *LHY*, *PRR9*, *GI*, and *TOC1* [40]. In *Arabidopsis* and *Drosophila*, PRMT5 (PROTEIN ARGININE METHYL TRANSFERASE 5) has also been implicated in regulating the splicing of circadian clock genes, most likely by modifying the methylation status of the Sm spliceosomal proteins [41,42]. More interestingly, PRMT5 is a clock-controlled gene [41], which suggests that the circadian clock might govern the alternative splicing of downstream genes through PRMT5. In contrast, the expression of both SKIP and STIPL1 showed no circadian rhythmicity. It will be intriguing to know whether other core splicing factors are under the circadian control or not.

The RNA-binding protein NONO (also called p54nrb in human and NonO in mouse) contains two RRM domains and a HTH domain, which suggests that it has dual DNA/RNA-binding activities. NONO is localized in the nuclear domains termed paraspeckles in the nucleolus [43,44], and it binds to the 3′end of U5 snRNA stem 1b, which is associated with both the spliceosome and U4/U6.U5 tri-snPNP, suggesting its role in splicing regulation [45]. The *N*-terminus of p54nrb/NonO exhibits high affinity to a set of RNA targets, including β-globin pre-mRNA, tumor necrosis factor αmRNA and the intronic pyrimidine-rich sequence in β-tropomyosin pre-mRNA [43,45].

Disruption of *nonA*, the *Drosophila* homolog of NONO, confers hyperactivity and mild arrhythmicity. Moreover, the mRNA expression of the clock gene *timeless* is significantly decreased and its rhythmicity is dampened by *nonA* disruption [45]. NONO can bind to the clock protein PER. Although NONO showed constant expression, its binding to clock protein PER1 displayed circadian rhythmicity [46]. In mice, loss of NONO leads to increased cell proliferation and decreased senescence. NONO binds to the p16-Ink4A cell cycle checkpoint gene and potentiates its circadian activation in a PER protein-dependent fashion, and lack of NONO results in defective wound repair [47,48]. NONO is also involved in cell cycle regulation by binding to PER; the two proteins act together as transcription factors for p16-Ink4A, which is a regulator of the mitogen-responsive retinoblastoma pathway and one of the key cellular components regulating senescence [48]. Depletion of either NONO or PER led to abolishment circadian expression of p16-Ink4A and elimination of the circadian cell cycle gating, suggesting that PER and NONO are responsible for the coupling of cell cycle to the circadian clock. However, these functions of NONO seem to play a role in transcriptionally regulating the expression of *p16-Ink4A*, instead of alternative splicing.

## 3. Control of mRNA Polyadenylation

The eukaryotic poly(A) tail length at an mRNA 3′ end is under cellular control throughout the lifespan of the mRNA. The regulation of mRNA poly(A) tail length likely serves two major purposes: to control mRNA translation and to control mRNA turnover [49]. Intriguingly, Kojima *et al*. recently reported that the mRNA polyadenylation pattern of a substantial part of the transcriptome is under circadian control [25]. The CPEB (cytoplasmic polyadenylation element-binding protein) proteins bind to cytoplasmic polyadenylation elements (CPEs) in mRNA 3′UTR regions and recruit the polyadenylation complex encompassing the cytoplasmic poly(A) polymerase GLD2 and other related proteins. GLD2 and related proteins promote polyadenylation-induced translation [50]. In mice liver, the mRNA levels of *Cpeb2*, *Cpeb4*, *Parn* and *Gld2* are under circadian control, and these genes have been proposed to be responsible for the rhythmicity of transcriptome-scale polyadenylation [25].

*Nocturnin* (*Noc*, also called *Ccrn4l* [*carbon catabolite repression 4-like*]) is a member of the CCR4 deadenylase family, and it encodes a putative deadenylase that degrades mRNA poly(A) tails. *Noc* is a clock-controlled gene with peak levels during the night [51–55]. The promoter region of *Noc* harbors an E-box, a cyclic AMP response element (CRE) and a photoreceptor-conserved element II (PCE II), all of which are under circadian control [52,56,57]. Noc is an important mediator of lipid metabolism, adipogenesis, glucose homeostasis, inflammation and osteogenesis [53,58]. In the sponge *Suberites domuncula*, Nocturnin regulates the deadenylation of the *glycogenin* mRNA, which encodes a key metabolic enzyme for carbohydrate/glycogen metabolism. *Nocturnin* levels are high in the dark and low in the light, while *glycogenin* shows an opposite diurnal expression pattern [59].

Nitric oxide (NO) is a signaling molecule that regulates a diverse group of physiological and pathophysiological activities in cardiovascular, nervous and immunological systems. iNOS (inducible nitric oxide synthase) is a member of the nitric oxide synthase enzyme family, and it catalyzes the synthesis of NO. iNOS is induced by immunostimulatory cytokines, bacterial products or infection [60]. In mice, depletion of Nocturnin results in the destabilization of the *iNOS* mRNA and the impairment of the nighttime peak profile of hepatic *iNOS* mRNA, which suggests that the *iNOS* mRNA might be a target of Noc [61]. It is possible that Nocturnin serves to deadenylate *iNOS* mRNAs, which further leads to destabilization.

In *Neurospora*, FRH (FRQ-interacting RNA helicase) plays diverse roles in the regulation of the circadian clock [62–68]. FRH is the counterpart of yeast Mtr4p/Dob1p, which encodes a DexH RNA helicase. In yeast, Mtr4p associates with the Trf4-Air2 heterodimer to form the TRAMP (Trf4/Trf5-Air1/Air2-Mtr4 Polyadenylation) complex, which is an activating cofactor for the exosome. TRAMP promotes the 3′–5′ exoribonuclease activity of the exosome, which is responsible for mRNA surveillance and turnover [69–72]. Knockdown of *frh* in *Neurospora* has been shown to result in a dramatically slow growth rate and abolishment of conidial rhythmicity [62]. FRQ and FRH form the FRQ-FRH complex (FFC), which binds directly to *frq* mRNA. In an *frh* knockdown strain, the *frq* transcripts are more stable and contain significantly longer poly(A) tails compared to wild type, which implies that FRH participates in the regulation of mRNA polyadenylation [63]. FRH might mediate mRNA poly(A) tail length through its partners Trf4 and Trf5 (Topoisomerase one-requiring function 4/5) that are non-canonical poly(A) polymerases [70,73,74], which remains to be further investigated.

Although genome-wide experiments have highlighted the significance of regulation of mRNA poly(A) tail length in the circadian clock, the detailed mechanisms remain unclear. It will be important to address whether and how these RNPs, including NOC and FRH, regulate the poly(A) tail length of clock gene transcripts. In addition, the regulation of polyadenylation occurs in both the nucleus and the cytoplasm, which has functional discrepancy and consequence. It is necessary to elucidate where these factors exert their functions.

## 4. mRNA Quality Control

Surveillance mechanisms take place in both the nucleus and cytoplasm to eliminate incorrectly processed transcripts and terminate erroneous gene expression [75,76]. Coupled with as well as following transcription, mRNAs undergo a complicated chain of processing that includes capping, splicing and polyadenylation. Aberrant RNAs can arise from any of these events.

In the nucleus, the exosome is an exoribonuclease complex responsible for rRNA and tRNA biogenesis as well as mRNA surveillance [69,70]. *Neurospora* FRH is the homolog of yeast Mtr4p, a component of the TRAMP complex, which serves as an activating cofactor of the exosome for the degradation of aberrant RNAs via the nuclear RNA surveillance pathway [69,70]. However, whether FRH mediates the elimination of aberrant clock gene transcripts remains unknown.

The nonsense-mediated decay (NMD) pathway is a translation-coupled mRNA surveillance system that typically degrades transcripts bearing premature termination codons (PTCs), thus preventing the translation of unnecessary or aberrant transcripts [77–79]. In addition to its role in quality control, NMD plays a less clear role in regulating the abundance of normal transcripts [80,81].

UPF1 is an RNA helicase that plays a central role in nonsense-mediated mRNA decay (NMD). In *Neurospora*, the mutant strain *period-6* (*prd-6*), which bears a mutation in *upf1*, exhibits a shortened circadian period (approximately 19 h) and an abnormal temperature compensation of the circadian clock [82].

In *Arabidopsis*, defects in NMD have been reported to cause impairment in development, seed size control and responses to stress, injury and viral infection [83,84]. In *Arabidopsis*, *At*GRP7 (glycine-rich RNA-binding protein) and *At*GRP8 are two nuclear RRM (RNA-binding motif) domain-containing RBPs, and *At*GRP7 is a clock gene that is also regulated by the circadian clock [85,86]. Overexpression of either *At*GRP7 or *At*GRP8 leads to the aberrant accumulation of *Atgrp7* or *Atgrp8* transcripts with PTCs in an NMD-dependent fashion [85–87]. *At*GRP7 exhibits a variety of physiological functions, including the promotion of floral transition, plant immunity, growth and stress tolerance. Both atGTP7 and atGRP8 are RNA-binding proteins with a single RRM domain and a glycine-rich *C*-terminus. *At*GRP7 controls mRNA processing, mRNA folding and transport of downstream genes, and it plays a role in the circadian output and the regulation of several transcripts involved in stress responses [88,89].

NMD regulates ~10% of all cellular mRNAs. In addition to its role in elimination of PTC-containing transcripts, NMD has been implicated in the regulation of other types of transcripts, for instance, mRNAs with upstream open reading frames in their 5′UTRs and mRNAs harboring extended 3′UTRs due to aberrant polyadenylation site usage [75]. Therefore, it is critical to elucidate how NMD affects the circadian clocks at the molecular level.

## 5. mRNA Nuclear Transport

Appropriately processed mRNAs are transported from the nucleus to the cytoplasm for subsequent translation, under the control of a series of RBPs and RNP complexes [90]. Cold-inducible RNA-binding protein (CIRP) is a glycine-rich RNA-binding protein harboring an RNA recognition motif (RRM), whose expression can be induced by a mild decrease in temperature [91,92]. In cultured fibroblasts, the rhythmicity of CIRP expression is controlled by temperature cycles instead of circadian oscillators. In a circadian clock, CIRP is necessary for the maintenance of high-amplitude circadian gene expression. CIRP binds to the mRNAs of a number of clock genes and clock-associated genes, including *CLOCK*, *Sirtuin 1* (*SIRT1*), *PERIOD 3* (*PER3*), *NCOR1* and *RORα*. It has been shown that in CIRP-deficient cells, the *Clock* mRNA abundance is only moderately changed in nuclei, while the CLOCK protein level is significantly reduced in cytoplasm [91], which suggests that CIRP mediates the export of clock transcripts. CIRP might also regulate the compensation of circadian amplitude at low temperatures in mammals.

FMR1 and FRH are another two proteins that might be potentially implicated in the regulation of transport of clock gene mRNAs.

Fragile X syndrome, resulting from loss-of-function mutations that abolish the expression of the X-linked gene FMR1 (*Fragile X mental retardation 1*, also called *DFXR*), is the most common form of inherited mental retardation in humans [19]. *FMR1* encodes an RNA-binding protein that contains two ribonucleoprotein K homology domains (KH domains) and an arginine- and glycine-rich domain (RGG box) [19]. FMR1 plays an important role in synaptogenesis and axonal arborization [93]. In addition, FRM1 has been implicated in the regulation of the circadian clock [93–96]. In *Drosophila*, flies homozygous for *dfmr1* deletion displayed erratically timed, short bouts of relatively high activity. It has also been reported that, in constant darkness, the expression of PER and TIM was delayed, and the amplitude of PER was reduced [93]. The expression of cAMP response element-binding protein (CREB), a known clock-controlled gene, was also reduced in *fmr1* mutants, which suggests a role for FRMR1 in regulating both circadian oscillation and output [94].

There are two FMR1 paralogs in mice, *fragile X related gene 1* and *2* (*FXR1* and *FXR2*), which exert their functions in mRNA transportation and translation [95]. *Fmr1/Fxr2* double knockout (KO) and *Fmr1* KO/*Fxr2* heterozygous mice exhibit arrhythmic activity in a light-dark (LD) cycle, and *Fmr1* or *Fxr2* KO mice display shorter periods of free-running locomotor activity in total darkness (DD) [95]. The expression levels of a number of clock components, including *Bmal1*, *Cry*, *Per1* and *Per2*, are significantly altered in these animals [95]. Whether FMR1 influences the circadian clock by regulating the transport of clock gene mRNAs remains to be determined.

In a yeast *mtr4* mutant, poly(A) + RNAs were shown to be retained in the nucleus, suggesting a role for Mtr4p in regulating mRNA transport [96]. It will be interesting to investigate whether FRH, the counterpart of Mtr4p in *Neurospora*, mediates the transport of clock gene mRNAs.

## 6. Translational Regulation

In mouse liver, 20% of soluble proteins are subject to circadian control [97]. However, half of these genes do not exhibit rhythmic steady-state mRNA levels, which suggests that translational control also contributes to the generation of gene expression rhythmicity [97,98]. Recently, it has been shown that the mRNAs, including Eif4e, Eif4g1, Eif4a2, Eif4b, Eif4ebp1 and Eif4ebp3, which encode factors involved in translation initiation, are under circadian control. Moreover, the phosphorylation of EIF4G, EIF4B, 4EBP1 and ribosomal protein (RP) S6 (RPS6) oscillate in a circadian fashion, which peaks during the night [98]. These findings suggest that the circadian clock controls ribosome biogenesis and gene expression at the translational level.

*Drosophila* eclosion and locomotor activity are controlled by its circadian clock. LARK negatively regulates eclosion; decreased LARK levels account for an early eclosion phenotype, while increased LARK levels correspond to a late eclosion phenotype [99]. *lark* encodes a protein with two RNA recognition motifs (RRMs) and a retroviral-type (RT) zinc finger domain. LARK has been recently found to associate with and stabilize fragile X mental retardation protein (FMRP). The two proteins act together to regulate eye development and circadian behavior. In *Drosophila*, it has been shown that overexpression of LARK conferred arrhythmicity and a reduction of *dfmr1* expression can mostly rescue the arrhythmic phenotype, which suggests that FMRP promotes LARK activity [100]. In mouse suprachiasmatic nuclei (SCN), a tissue, which is an autonomous circadian pacemaker, the level of LARK protein oscillates with a similar phase to PER1 protein although its mRNA shows no rhythmicity [101]. LARK post-transcriptionally regulates *Per1* expression by binding to the 3′UTR of *Per1* mRNA, which further promotes the synthesis of PER1 protein. Knockdown of Lark resulted in short circadian period while overexpression of *Lark* resulted in prolonged period, which suggests that LARK might regulate circadian clock by controlling the translation of *Per1*.

*Rev-erb α* encodes a transcriptional repressor in the positive limb of circadian transcription (Figure 1), which harbors an internal ribosomal entry site (IRES) in its mRNA 5′UTR region [102]. IRES regulates the translation by recruiting ribosomes in a cap-independent manner. PTB is a RNA-binding protein which plays roles in diverse cellular processes, including polyadenylation, mRNA stability and translation initiation. hnRNP Q is a member of hnRNP family, which function to bind pre-mRNAs and facilitate their processing of mRNAs. *Rev-erb α* mRNA has an internal ribosomal entry site (IRES) in its 5′-UTR, which can be bound by PTB and hnRNP Q; PTB and hnRNP Q function to enhance the translation of *Rev-erb α* through the IRES element [102]. hnRNP Q is also an IRES trans-acting factor of mouse clock gene *Period 1* (*Per1*), and the binding between mhnRNP Q and *mPer1* mRNA is in a rhythmic fashion. Knockdown of mhnRNP Q caused a decrease in PER1 protein levels and a slight delay in mPER1 expression without change in mRNA abundance [103].

hnRNP Q modulates the translation of AANAT protein levels in a similar way. Serotonin *N*-acetyltransferase (arylalkylamine *N*-acetyltransferase (AANAT) is the key enzyme in melatonin synthesis regulated by the circadian clock and *AANAT* mRNA contains an IRES element within its 5′UTR. The 68-kDa hnRNP Q binds specifically to AANAT IRES element; hnRNP enhances the translation and contributes to AANAT rhythmicity at the protein level [104]. These data suggest that hnRNP Q plays an important role in translational control of melatonin synthesis. In addition, hnRNP Q is a spliceosomal component, however, whether hnRNP Q mediates mRNA splicing of clock genes remains elusive.

Bioluminescence is generated by a luciferase-catalyzed reaction involving the substrate luciferin. Binding and stabilization of luciferin by luciferin-binding protein (LBP) is also necessary for the bioluminescence. The bioluminescence in *Gonyaulax polyedra* is controlled by circadian clock, which displays a night phase *in vivo*. In contrast to its constant mRNA level, the LBP protein abundance oscillates between day and night with a 10-fold circadian variation [105]. Circadian-controlled translational regulator (CCTR) rhythmically binds to *lbp* mRNA at the 22-nt UG repeat-containing region of its 3′UTR and represses the translation of target transcripts [106]. In the green alga *Chlamydomonas reinhardtii*, clock-controlled RNA-binding protein (CHLAMY 1) is an analog of *Gonyaulax* CCTR. CHLAMY 1 binds specifically to the 3′UTRs of several mRNAs and recognizes them all via a common *cis*-acting element [107]. LBP is an example that evidences the role of translational control in generating the circadian rhythmicity.

The ribosome is a large ribonucleoprotein complex that functions as the translational machinery of the cell, and translation efficiency is essential for controlling protein translation, conformation and function [108]. The translation rate depends on the codon usage since it is more efficient for the transfer RNA (tRNAs) to recognize preferential codons and carry the corresponding amino acid to the ribosome for peptide synthesis. The process of translation of certain clock proteins is strictly controlled by the genetic codon usage. Two independent studies recently revealed that in *Neurospora* and cyanobacteria, non-preferential codon usage for the clock genes (*frq* in *N. crassa* and *KaiBC* in *S. elongatus*) determines the translation, conformation or function of the clock proteins [109,110]. Optimization of the genetic codon usage in *Neurospora frq* gene, which uses non-optimal codons resulted in severe impairment of the conidiation rhythmicity. More interestingly, it led to changes in phosphorylation profile, conformation and function of FRQ protein [109]. In the cyanobacterium *S. elongates*, optimization of *KaiBC* genes resulted in promoted amplitude at low temperatures. However, at low temperatures, the strain with optimization of *KaiBC* exhibits a slower growth rate [110], which suggests that codon usage is essential for the adaption to the ambient conditions. In humans, the synonymous substitution polymorphism T2434C in the clock gene *PER1* has been reported to be associated with extreme diurnal preference. C2434 was more frequently found in subjects with extreme morning preference than in subjects with extreme evening preference [111]. Whether the T2434C polymorphism affects mRNA stability or translation rate is unknown.

## 7. Regulation of mRNA Turnover

The steady-state level of mRNA in a cell is determined by both its production and lifespan. Regulation of mRNA turnover is a critical event during post-transcriptional processing as alteration in mRNA abundance may change the amount of the corresponding protein. mRNA turnover is controlled by a series of factors in a stepwise fashion, encompassing decapping, deadenylation and degradation [112].

In the cytoplasm, the exosome complex regulates the 3′–5′ degradation of mRNAs. Of the 10 subunits of the exosome, RRP44/Dis3 is the catalytic subunit with nuclease activity [113,114]. In *Neurospora*, knockdown of *rrp44* resulted in stabilization of *frq* mRNA. In addition, RRP44 regulates the expression of a set of clock-controlled genes either directly or indirectly. The conidial rhythmicity was abolished and the longer circadian periods of *frq* mRNA and FRQ protein were significantly longer in *rrp44-*silenced strain [63].

AU-rich elements (AREs) are located in the 3′UTR of many mRNAs and found in short-lived transcripts of a diverse group of genes, such as cytokines, proto-oncogenes, growth factors, and cell cycle regulators [115]. AREs generally serve as binding sites for AU-binding proteins (AUBPs), which cause deadenylation, decapping and degradation of the target transcripts. Therefore, AUBPs are known to have mRNA decay-promoting properties. Butyrate response factor-1 (BRF1, also called ZFP36L1/Tis11b) is an AUBP that possesses two zinc-finger domains in tandem [116]. BRF1 is expressed in a circadian fashion [116], which suggests that it might be involved in the post-transcriptional regulation of gene expression rhythmicity. Analogously, although the *Neurospora frq* gene contains no canonic ARE, the 3′UTR region of *frq* determines its transcript decay rate [63], both cis-elements and trans-factors might be involved in regulating the decay of *frq* mRNA. hnRNP proteins are involved in the regulation of mRNA stability. In mammals, hnRNP Q, hnRNP L and hnRNP R bind to *AANAT* mRNA 3′UTR region and destabilize the transcripts; hnRNP Q, hnRNP L and hnRNP R are necessary for maintaining the normal rhythm of melatonin [117]. hnRNP Q also binds to both the mRNA 5′ and 3′ UTR regions of mouse clock gene *Period3*, to regulate the translation and mRNA decay respectively [118]. There are several known pathways that control the mRNA turnover, which are 3′→5′ exonucleolytic decay, 5′→3′ exonucleolytic decay and endonuclease cleavage, respectively [112,115]. It will be critical to elucidate the following pathways coupled to hnRNP proteins to regulate the turnover of clock gene transcripts.

miRNAs (micro RNAs) are small non-coding RNAs with a length of approximately 22 nucleotides that trigger gene silencing. miRNA-mediated gene silencing is an important mechanism of gene expression regulation in many eukaryotes through facilitation of mRNA decay or translational repression [119,120]. Dicer, an RNase III-type endonuclease, and RISC (RNA-induced silencing complex) are two essential RNP complexes required for miRNA-mediated gene silencing [121]. A number of miRNAs have been implicated in the regulation of circadian clocks in different species [122–128]. It is reasonable to speculate that some miRNAs might be responsible for the generation of circadian rhythmicity at the protein level of a set of genes that are constantly expressed at the mRNA level.

## 8. Summary and Outlook

**Figure 2:**
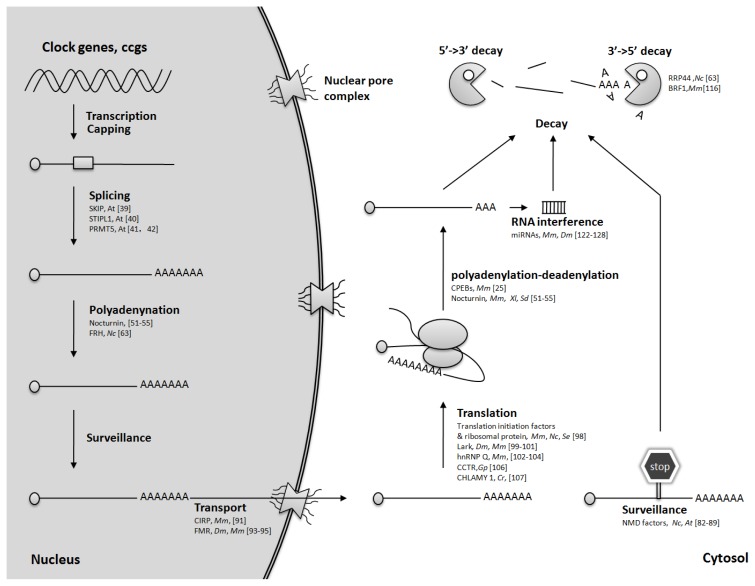
Known RNP factors involved in stepwise controlling of expression of clock genes on the mRNA level. The species names are abbreviated as Nc: *Neurospora crassa*; Mm: mammals; Dm: *Drosophila melanogaster*; At: *Arabidopsis thaliana*; Cr: *Chlamydomonas reinhardtii*; Gp: *Gonyaulax polyedra*; Se: *Synechococcus elongates*; Sd: *Suberites domuncul*a; Xl: *Xenopus laevis*.

Eukaryotic genomes encode a large number of RBPs that are involved in the regulation of a variety of biological processes [17,19]. Increasing data have demonstrated that RNP complexes play multiple roles in regulating the expression of circadian clock genes (Figure 2). However, little is known about the roles of RNP complexes in certain steps of mRNA processing, such as mRNA capping/decapping and transport. Post-transcriptional steps are tightly coupled and interwoven, which suggests that ribonucleoprotein complexes constitute highly complex regulatory networks coordinating to sustain circadian clock systems. Some proteins, like hnRNP Q, FRH and NONO, appear to be versatile players in multiple post-transcriptional regulations. Relative to the accumulation of evidence suggesting the influence on the circadian phenotypes, the functional details of these RNP/RNP complexes underlying the circadian clocks remain to be intensively investigated. Therefore, it is of significant importance to systematically identify more clock-associated RNPs and elucidate their roles. With the cutting edge technologies, we will be able to identify more RNPs involved in circadian clock and target transcripts in a large scale.

It is not surprising that these networks might be involved in temperature compensation, which is an important property of any circadian clock [30]. Moreover, comprehensive modulation of circadian clocks by nucleoprotein complexes is likely to account for the fine regulation of the circadian systems under various conditions and stresses. Further investigation of the RNP roles in these issues will expand our knowledge of both the circadian clock mechanisms and the physiological relevance of circadian clocks. In addition, studies of the roles of SNPs in circadian clocks will no doubt help in revision of the basic model of the generation of circadian rhythms.
